# A simple method for estimating relative risk using logistic regression

**DOI:** 10.1186/1471-2288-12-14

**Published:** 2012-02-15

**Authors:** Fredi A Diaz-Quijano

**Affiliations:** 1 Grupo Latinoamericano de Investigaciones Epidemiológicas, Organización Latinoamericana para el Fomento de la Investigación en Salud (OLFIS), Bucaramanga, Colombia

**Keywords:** Logistic regression, Odds ratio, Prevalence ratio, Relative risk.

## Abstract

**Background:**

Odds ratios (OR) significantly overestimate associations between risk factors and common outcomes. The estimation of relative risks (RR) or prevalence ratios (PR) has represented a statistical challenge in multivariate analysis and, furthermore, some researchers do not have access to the available methods. Objective: To propose and evaluate a new method for estimating RR and PR by logistic regression.

**Methods:**

A provisional database was designed in which events were duplicated but identified as non-events. After, a logistic regression was performed and effect measures were calculated, which were considered RR estimations. This method was compared with binomial regression, Cox regression with robust variance and ordinary logistic regression in analyses with three outcomes of different frequencies.

**Results:**

ORs estimated by ordinary logistic regression progressively overestimated RRs as the outcome frequency increased. RRs estimated by Cox regression and the method proposed in this article were similar to those estimated by binomial regression for every outcome. However, confidence intervals were wider with the proposed method.

**Conclusion:**

This simple tool could be useful for calculating the effect of risk factors and the impact of health interventions in developing countries when other statistical strategies are not available.

## Background

The odds ratio (OR) is commonly used to assess associations between exposure and outcome and can be estimated by logistic regression, which is widely available in statistics software. OR has been considered an approximation to the prevalence ratio (PR) in cross-sectional studies or the risk ratio (RR, which is mathematically equivalent to PR) in cohort studies or clinical trials. This is acceptable when the outcome is relatively rare (< 10%). However, since many health outcomes are common, the interpretation of OR as RR is questionable because OR overstates RR, sometimes dramatically [1-3]. Moreover, the OR has been considered an "unintelligible" effect measure in some contexts [3].

Binomial regression has been recommended for the estimation of RRs (and PRs) in multivariate analysis [4]. However, sometimes this statistical method cannot estimate RR because convergence problems are frequent. Therefore, the Cox regression with robust variance has been recommended as a suitable method for estimating RRs [5,6].

However, these statistical methods (binomial and Cox regression) are not widely available in freeware (such as Epidat or Epi-Info). Therefore, the ability to estimate PRs and RRs in multivariate models could be limited in research groups with scant resources. In this article, a strategy for estimating RRs with ordinary logistic regression is proposed. This new method could be useful for identifying risk factors and estimating the impact of health interventions in developing countries.

## Methods

### Database

A database of 1000 observations with dichotomous variables was created to simulate a cohort study in which a common event (incidence of 50%) would be strongly related to two independent predictors (*A *and *B*). These predictors would also be statistically associated with one another, resulting in a moderate confounding effect. Then, a third independent variable with a prevalence of 40% was included (predictor *C*). This variable was randomly distributed, but more often in positive than negative predictor *A *group. Thus, this variable was statistically associated with the outcome in a univariate analysis but the association would be explained by the presence of predictor *A *in a multivariate model. Finally, additional dependent variables were generated by randomly selecting a proportion of cases. Thus, outcome variables with frequencies of 20% and 5% were obtained. The first table shows the hypothetical distribution of subjects according to the predictors and outcomes (Table 1).

**Table 1:** Hypothetical distribution of subjects according to the predictors and outcome incidence

	High incidence (50%)		Intermediate incidence (20%)		Low incidence (5%)		
		
Independent Variable	Cases (n = 500)	Non-cases (n = 500)	Cases (n = 200)	Non-cases (n = 800)	Cases (n = 50)	Non-cases (n = 950)	Total (n = 1000)
**Predictor A**							
positive	409	191	161	439	45	555	600
negative	91	309	39	361	5	395	400

**Predictor B**							
positive	398	102	159	341	36	464	500
negative	102	398	41	459	14	486	500

**Predictor C**							
positive	227	173	84	316	23	377	400
negative	273	327	116	484	27	573	600

### Statistical analysis

Statistical analysis was performed using STATA software (STATA^®^/IC 11.0). RRs and 95% confidence intervals (CI) were estimated by applying log-binomial regression and Cox regression with a constant in the time variable [6]. In order to obtain corrected CIs by Cox regression, the robust variance option was applied [7]. ORs and their correspondent CIs were also estimated using an ordinary logistic regression. After univariate estimations were calculated, ORs and RRs were obtained in multivariate models including all independent variables (predictors A, B and C).

### Proposed modification to logistic regression analysis

The log-binomial model is similar to logistic regression in assuming a binomial distribution of the outcome. However, in a logistic regression the link function is the logarithm of the odds, which is the ratio between cases and non-cases, while in binomial regression the link function is the logarithm of the proportion, i.e., the ratio between cases and cases plus non-cases [4].

In a binomial regression model with k covariates, the function is written as:

$$\text{Log }\left[\text{a}/\left(\text{a}+\text{b}\right)\right]\;=\beta_{0}+\;\beta_{1}X_{1}+\ldots +\beta_{k}X_{k}$$

where *a *is the number of cases and *b *is the number of non-cases, and *X_i _*the covariates. Thus, a/(a + b) is the probability of success (e. g., the proportion of sick persons in a group), and the RR (or PR) estimated of a given covariate X_i _is *e*^βi^.

On the other hand, in a logistic regression model, the function is written as:

$$\text{Log }\left(\text{a}/\text{b}\right)\;=\beta_{0}+\;\beta_{1}X_{1}+\;\ldots \;+\;\beta_{k}X_{k}$$

where a/b is the odds of success and the OR estimated of a given covariate X_i _is *e*^βi^.

In order for the case information to be included in the denominator of the estimates in a logistic regression, all observed cases were duplicated in a provisional database and identified as *non-cases*. Thus, a number of observations was included equaling that of the cases and containing the same information about the covariates. Thus, this new logistic function could be written as:

$$\text{Log }\left[\text{a}/\left(\text{y}\right)\right]\;=\beta_{0}+\;\beta_{1}X_{1}+\;\ldots \;+\;\beta_{k}X_{k}$$

where *y *includes non-cases as well as cases, although all of them are identified as non-cases. Afterwards, a logistic regression procedure was performed with the modified dataset. The "ORs" obtained were considered direct estimations of RRs because β_i _defined the relationship between X_i _and the Log [a/(y)], which in this model would be mathematically similar to Log [a/(a + b)] of the log-binomial model. For each outcome, a provisional database was prepared.

This strategy for logistic regression recognizes an entire cohort as controls. This trick is innovative but analogous to the analysis of case-cohort studies. In that design, cases of a particular outcome are compared with a sample (sub-cohort) of the entire cohort that gave rise to all cases [8]. The objective of selecting this sub-cohort is to estimate the frequency of exposure in the entire cohort. For this reason, such studies have also been called case-exposure studies [9].

This sub-cohort may include some cases, which would consequently be overrepresented in the analysis. Then, by comparing the frequency of exposure between the cases and the sub-cohort set, we obtain a direct estimate of RR (not OR) [9-11]. Similarly, in the method proposed here, the cases would be compared against the entire cohort and thus all cases would be overrepresented. This affects the variance of the estimates and for this reason the CIs are wider [11]. Therefore, an inflation factor for the Standard Error (SE) of each predictor and outcome incidence was calculated as the ratio between SE obtained with the proposed method and SE resulting from binomial regression (as reference method).

## Results

For the rarer event (incidence of 5%), RRs estimated by log-binomial were similar to those calculated both by the Cox regressions and the proposed method (modified logistic regression) (Table 2). Few differences were identified among the CIs of RRs: CIs from the modified method were wider than those estimated by log-binomial and Cox regression with the robust variance option. ORs estimated by ordinary logistic regression were close to RR values. Predictors *A *and *B *were statistically associated with the outcome in univariate analysis but only *A *was independently associated in the multivariate model (Table 2).

**Table 2:** RRs and ORs and corresponding CIs of associations between a rare event (incidence = 5%) and three independent variables, estimated by Log-binomial regression, ordinary logistic regression, Cox regression with robust variance and logistic regression with the proposed modification

Independent variable	Log-binomial regression: RR (CI)	Logistic regression: OR (CI)	Cox regression - robust: RR (CI)	Modified Logistic regression: RR (CI)
**Predictor *A***				
Unadjusted	6 (2.4 - 14.98)	6.41 (2.52 - 16.28)	6 (2.4 - 14.99)	6 (2.36 - 15.25)
Adjusted *	4.96 (1.89 - 12.98)	5.26 (1.97 - 14.06)	4.97 (1.91 - 12.92)	4.99 (1.86 - 13.34)

**Predictor *B***				
Unadjusted	2.57 (1.4 - 4.71)	2.69 (1.43 - 5.06)	2.57 (1.4 - 4.71)	2.57 (1.37 - 4.83)
Adjusted *	1.59 (0.85 - 2.97)	1.64 (0.85 - 3.18)	1.59 (0.84 - 3.01)	1.59 (0.82 - 3.09)

**Predictor *C***				
Unadjusted	1.28 (0.74 - 2.2)	1.29 (0.73 - 2.29)	1.28 (0.74 - 2.2)	1.28 (0.72 - 2.26)
Adjusted *	0.98 (0.57 - 1.69)	0.97 (0.54 - 1.74)	0.97 (0.57 - 1.65)	0.96 (0.54 - 1.72)

* Adjusted by the other independent variables.

For the second and third outcomes, with incidences of 20% and 50% respectively, the differences between RRs in log-binomial regression and ORs in ordinary logistic regression were more evident (Tables 3 and 4). This was especially remarkable for the commonest event, where the ORs of predictors A and B were at least twice the corresponding RR values (Table 4).

**Table 3:** RRs and ORs and corresponding CIs of associations between an intermediate frequency event (incidence = 20%) and three independent variables, estimated by Log-binomial regression, ordinary logistic regression, Cox regression with robust variance and logistic regression with the proposed modification

Independent variable	Log-binomial regression: RR (CI)	Logistic regression: OR (CI)	Cox regression - robust: RR (CI)	Modified Logistic regression: RR (CI)
**Predictor A**				
Unadjusted	2.75 (1.99 - 3.81)	3.39 (2.33 - 4.95)	2.75 (1.99 - 3.81)	2.75 (1.9 - 3.99)
Adjusted *	1.79 (1.27 - 2.52)	2.06 (1.36 - 3.12)	1.77 (1.26 - 2.48)	1.75 (1.16 - 2.64)

**Predictor B**				
Unadjusted	3.88 (2.82 - 5.34)	5.22 (3.6 - 7.56)	3.88 (2.82 - 5.34)	3.88 (2.69 - 5.59)
Adjusted *	3.15 (2.24 - 4.43)	4.07 (2.75 - 6.03)	3.15 (2.26 - 4.39)	3.15 (2.13 - 4.65)

**Predictor C**				
Unadjusted	1.09 (0.85 - 1.4)	1.11 (0.81 - 1.52)	1.09 (0.85 - 1.4)	1.09 (0.8 - 1.48)
Adjusted *	0.92 (0.72 - 1.17)	0.89 (0.63 - 1.25)	0.92 (0.72 - 1.17)	0.93 (0.67 - 1.28)

* Adjusted by the other independent variables.

**Table 4:** RRs and ORs and corresponding CIs of associations between a common event (incidence = 50%) and three independent variables, estimated by Log-binomial regression, ordinary logistic regression, Cox regression with robust variance and logistic regression with the proposed modification

Independent variable	Log-binomial regression: RR (CI)	Logistic regression: OR (CI)	Cox regression - robust: RR (CI)	Modified Logistic regression: RR (CI)
**Predictor *A***				
Unadjusted	3 (2.48 - 3.62)	7.27 (5.44 - 9.72)	3 (2.48 - 3.62)	3 (2.31 - 3.89)
Adjusted *	1.9 (1.58 - 2.28)	4.07 (2.88 - 5.74)	1.89 (1.56 - 2.28)	1.88 (1.41 - 2.51)

**Predictor *B***				
Unadjusted	3.9 (3.26 - 4.67)	15.23 (11.19 - 20.71)	3.9 (3.26 - 4.67)	3.9 (3.04 - 5.01)
Adjusted *	3.08 (2.56 - 3.72)	10.97 (7.95 - 15.14)	3.09 (2.56 - 3.72)	3.09 (2.36 - 4.04)

**Predictor *C***				
Unadjusted	1.25 (1.1 - 1.41)	1.57 (1.22 - 2.03)	1.25 (1.1 - 1.41)	1.25 (1 - 1.55)
Adjusted *	1.02 (0.95 - 1.1)	1.12 (0.8 - 1.57)	1.05 (0.96 - 1.15)	1.06 (0.84 - 1.34)

* Adjusted by the other independent variables.

**Figure 1:**
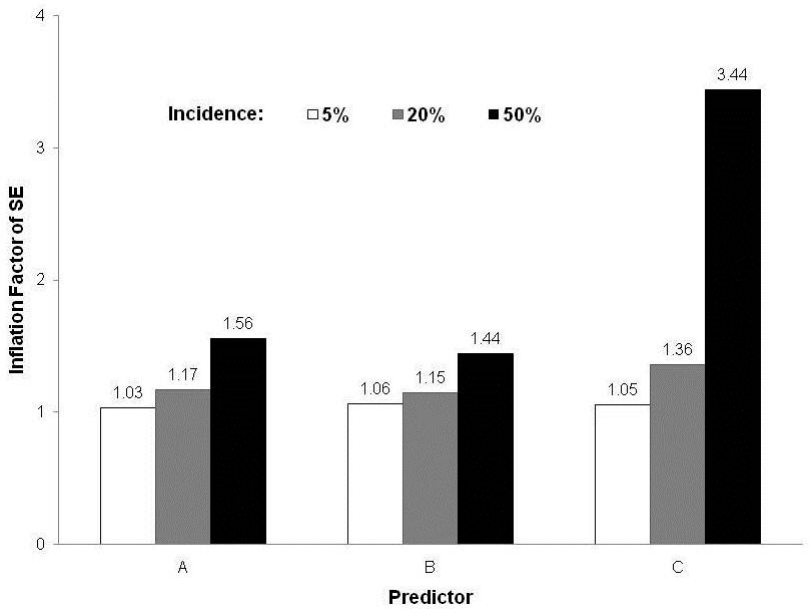
**Inflation Factor of Standard Error (SE) for each predictor according to incidence of outcome**.

On the other hand, RRs estimated in Cox regressions and modified logistic regression were similar or virtually identical to those estimated by log-binomial regression. However, the CIs outputted by the proposed method were wider than those obtained by the other models (Tables 3 and 4). Consequently, the SE inflation factor rose for each predictor as the outcome frequency increased (Figure 1).

## Discussion

The use of an adjusted odds ratio to estimate an adjusted relative risk or prevalence ratio is appropriate for studies of rare outcome but may be misleading when the outcome is common. Such overestimation may inappropriately affect clinical decision-making or policy development [3]. For example, overestimation of the importance of a risk factor may lead to unintentional errors in the economical analysis of potential intervention programs or treatment, which could be particularly harmful in developing countries.

The ordinary logistic model estimates OR (not RR) and was initially adapted for case-control studies since data from this type of study design can only determine OR [12]. Moreover, a case-control study is an optimal choice for analyzing rare-event risk factors, for which OR is a close approximation of RR. Thus, ordinary logistic regression is eminently useful for case- control studies mainly because the numeric value of OR mimics RR [12].

On the other hand, RR and PR can be directly determined from data based on cohort and cross-sectional studies, respectively, which are practical only for relatively common outcomes. However, in such circumstances OR estimated by ordinary logistic regression will be more discrepant than RR (or PR). This was exemplified in the results of this paper in that ORs progressively overestimated RRs as the outcome frequency increased.

Indeed, OR will always be greater than RR if RR is greater than 1 (adverse event) and OR will also be less than RR if RR less than 1 (protective effect). Therefore, the uncritical application of logistic regression and the misinterpretation of OR as RR can lead to serious errors in determination of both the importance of risk factors and the impact of interventions on clinical practice and public health [13].

For these reasons, several strategies for estimating RRs in multivariate analysis have been proposed [7,14-16]. Binomial regression is considered the most adequate choice. However, binomial models often predict probabilities greater than *one *and sometimes this regression cannot find possible values and converge in a model. Consequently, other alternative methods have been proposed when binomial regression cannot converge in a model. Cox regression with robust variance using a constant in the time variable seems like a good alternative [7]. However, these options and other statistical alternatives are only available in sophisticated software that some research groups cannot afford.

This paper presents a strategy for logistic regression that recognizes an entire cohort as controls. As the results show, this method can appropriately estimate RRs or PRs, even in analyses with common outcomes. Moreover, the method proposed in this article could be easily performed using free statistics programs that include only logistic regression for multivariate analysis of dichotomous outcomes.

However, the proposed method is associated with SE inflation, which increases confidence intervals. A simple and practical correction factor cannot be established for this problem because, in a multivariate regression, the standard error for each predictor depends on its correlation with all variables included in the model.

Therefore, since the obtained CIs can be wider than those estimated by other models, investigators must be aware that the risk of Type II error could be higher. For this reason, when an association is not statistically significant with the proposed method, ordinary logistic regression could be used for testing the hypothesis that association measure is different than unity. This is possible since the null hypothesis is mathematically equivalent for both OR and RR, because when RR is equal to 1, OR is also equal to 1.

## Conclusion

The proposed method may be useful for estimating RRs or PRs appropriately in analysis of common outcomes. However, because the resultant CIs are wider than those derived from other methods, this strategy should be employed when logistic regression is the only method available. This new method may help research groups from developing countries where access to sophisticated programs is limited.

## Abbreviations

CI: Confidence interval; OR: Odds ratio; PR: Prevalence ratio; RR: Relative risk; SE: Standard Error

## Competing interests

The author declares that they have no competing interests.

## Authors' contributions

FAD conceived the study, created the database, designed and executed the analysis, and wrote the manuscript.

## Pre-publication history

The pre-publication history for this paper can be accessed here:

http://www.biomedcentral.com/1471-2288/12/14/prepub
